# A Comparative Study to Evaluate the Efficacy of Dexmedetomidine and Clonidine to Accentuate the Perioperative Analgesia of Caudal 0.25% Isobaric Levobupivacaine in Pediatric Infraumbilical Surgeries

**DOI:** 10.7759/cureus.27825

**Published:** 2022-08-09

**Authors:** Kanta Bhati, Nitish Saini, Neha Aeron, Sonali Dhawan

**Affiliations:** 1 Anaesthesia, Sardar Patel Medical College and Prince Bijay Singh Memorial Hospital, Bikaner, IND; 2 Anaesthesia, Sarder Patel Medical College, Bikaner, IND

**Keywords:** caudal anesthesia, α2 agonists, levobupivacaine, pediatrics, pain

## Abstract

Background

Caudal block is an efficient way to offer perioperative analgesia for painful sub-umbilical interventions. It enables early ambulation and periprocedural hemodynamic stability. These are important advantages over general anesthesia, notably in preterm babies and in children with cardiopulmonary comorbidities. In this study, we aimed to compare the effect of dexmedetomidine and clonidine to accentuate the perioperative analgesia of 0.25% isobaric levobupivacaine in pediatric caudal anesthesia.

Methodology

A prospective double-blind randomized control study was conducted on 60 patients with the American Society of Anesthesiologists (ASA) physical status I, between the ages of one to six undergoing infraumbilical abdominal surgery under caudal anesthesia in the Department of Anaesthesia, Sardar Patel Medical College, Bikaner. Patients were randomly allocated to the following three groups of 20 each: group L, 1 mL/kg of levobupivacaine 0.25%; group LD, 1 mL/kg of levobupivacaine 0.25% with 0.5 µg/kg of dexmedetomidine; and group LC, 1 mL/kg of levobupivacaine 0.25% with 0.5 µg/kg of clonidine. Intraoperative and postoperative hemodynamic parameters were recorded for 24 hours. Patients’ pain scores, sedation scores, and Bromage scores were recorded. In our study, the main observation was the duration of analgesia and the total analgesic requirement for 24 hours.

Results

There was a significant difference in the duration of analgesia among the three groups (p < 0.001). Group LC had the highest duration of analgesia of 492.00 (50.01) minutes, followed by group LD 486.00 (54.71) minutes, and the lowest in group L 291.00 (40.25) minutes. There was a significant difference between the three groups in terms of the total dose of analgesics in 24 hours (p < 0.001), with the median total dose of analgesics being the highest in Group L. Three groups differed significantly in terms of motor block, which was limited to up to 180 minutes in groups LC and LD with no residual motor block.

Conclusions

The addition of α2 agonists such as clonidine or dexmedetomidine at a dose of 0.5 µg/kg as an adjuvant to caudal levobupivacaine (0.25%) at 1 mL/kg significantly prolongs the duration of opioid-free analgesia in children undergoing infraumbilical abdominal surgeries without prolonging the motor blockade and any side effects. Moreover, dexmedetomidine does not offer a significant advantage over clonidine regarding the analgesia duration.

## Introduction

Regional anesthesia is an essential part of modern pediatric anesthetic practice, conveying significant advantages to the patient and the hospital [1]. Caudal anesthesia is a safe and efficient way to offer perioperative analgesia and anesthesia in pediatric patients undergoing sub-umbilical surgeries. Perioperative hemodynamic stability, early ambulation, and avoidance of airway manipulation-related complications are known advantages of caudal anesthesia over general anesthesia [2].

The safety profile of caudal block has considerably improved with the advent of local anesthetics such as levobupivacaine and ropivacaine. Levobupivacaine, (-) enantiomer of bupivacaine, has efficacy equivalent to bupivacaine [3].

The addition of adjuvants modifies the onset time, efficacy, and duration of local anesthetics. Clonidine is an α2-adrenoceptor agonist. Studies on children have shown that caudal clonidine increases the duration of postoperative analgesia without respiratory depression [4]. Dexmedetomidine has an eight-fold greater affinity for α2 adrenergic receptors than clonidine. A major advantage of dexmedetomidine is its higher selectivity for α2A receptors which are responsible for the hypnotic and analgesic effects [5]. Studies have shown 1-2 µg/kg as an effective dose for caudal anesthesia.

Though many studies have been performed on effective doses of α2 agonists for accentuating the effect of local anesthetics, studies on low-dose adjuvants are lacking. The aim of this randomized, prospective, double-blind study was to compare the effect of low-dose dexmedetomidine and clonidine to accentuate the perioperative analgesia of 0.25% isobaric levobupivacaine in pediatric caudal anesthesia undergoing lower abdominal surgery.

## Materials and methods

This study was conducted after obtaining approval from the Institutional Ethics Committee at Sardar Patel Medical College and A.G. of Hospitals from July 2021 to December 2021. Written informed consent from the patient’s parent or caretaker was obtained. In total, 60 patients with the American Society of Anaesthesiologists (ASA) physical status I, aged one to six years of both sexes, scheduled for infraumbilical elective surgery were prospectively enrolled in this study.

Exclusion criteria included emergency surgery; known allergy to study drugs; known history of active renal, hepatic, respiratory, or cardiac disease; a history of seizures, neurological, or neuromuscular disorder; known or suspected coagulopathy, refusal, cutaneous local infection, or any congenital malformation.

**Figure 1:**
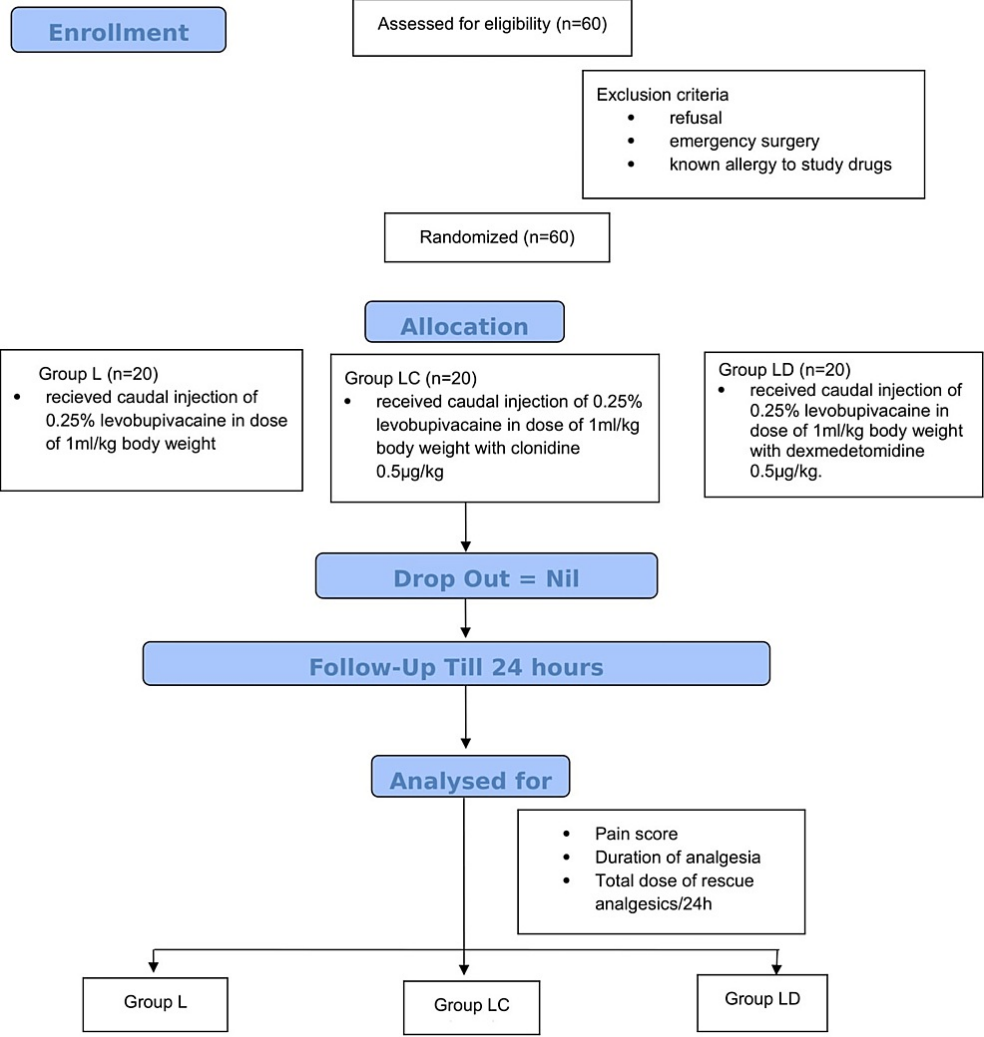
Consort diagram.

Children enlisted for the study were randomly allocated into three groups using a computer-generated randomization chart. Patients in group L (n = 20) received caudal injection of 0.25% levobupivacaine at a dose of 1 mL/kg body weight, patients in group LC (n = 20) received caudal injection of 0.25% levobupivacaine at a dose of 1 mL/kg body weight with clonidine 0.5 µg/kg, and patients in group LD (n = 20) received caudal injection of 0.25% levobupivacaine at a dose of 1 m:/kg body weight with dexmedetomidine 0.5 µg/kg (Figure 1).

After obtaining the patient’s weight and according to the randomizing table, the volume of each local anesthetic solution was prepared by one anesthetist in a coded transparent 10 mL syringe and labeled with the patient’s study number. All healthcare personnel, parents and guardians, and the anesthetist who performed the block were blinded to the caudal medications administered.

All patients fasted for six hours for solid food and two hours for clear fluids. All patients received preoperative midazolam at a dose of 0.5 mg/kg per orally (injectable midazolam mixed with apple juice) 20-30 minutes before the procedure in the preoperative area under monitoring. At the time of premedication, topical anesthetic cream (EMLA, eutectic mixture of local anesthetics: Lignocaine & Prilocaine) was applied to the site of intended needle puncture and potential sites of venepuncture.

On arrival at the operating theater, the standard monitors including a non-invasive blood pressure monitor and pulse oximeter were attached to the patient. An intravenous line with a 22-gauge/24-gauge cannula was secured and injection Isolyte-P was started at a rate of 5-10 mL/kg/hour. The patient was preoxygenated with 100% oxygen, and a sedative dose of injection ketamine 1 mg/kg intravenous along with sevoflurane (4% in oxygen at 6 L/minute via a face mask) was given for providing immobility during the procedure. The child was put in a lateral position with the knee flexed 90 degrees. The sacrococcygeal ligament was palpated between the two sacral cornua, and under aseptic precautions, a sterile 22-gauge needle was used to penetrate the skin at an approximate 45 degree angle. The penetration of the ligament was confirmed by pop sensation. Once the ligament has been passed, a flatter angle was adjusted by descending the needle before it was advanced to final position. After cautious aspiration for blood and cerebrospinal fluid (CSF), the allocated drug was injected slowly over the 60s.

After performing caudal block, the patient was turned to a supine position and anesthesia was maintained with sevoflurane in oxygen at 6 L/minutes through a face mask or by using a laryngeal mask airway if needed. No muscle relaxants and no further additional analgesics were given during the intraoperative period. Surgery was allowed after achieving the sensory block at T8 level which was assessed by the change in heart rate using the pin prick method or after a delay of at least 15 minutes between caudal blockade and surgical incision. The block was considered successful if there was no dorsiflexion at the ankle joint or flexion at the knee joint; no hemodynamic changes to the skin incision (absence of an increase in systolic blood pressure (SBP) or heart rate (HR) by >15% of pre-incision baseline values). Mean arterial pressure (MAP) and HR were observed just after the caudal block and then after five, 15, and 30 minutes intraoperatively. Minimum alveolar concentration (MAC) was recorded at 15 minutes post-block interval intraoperatively. At the end of the surgery, the patient was shifted to the post-anesthesia care unit (PACU) with stable vitals.

Postoperatively, pain score, motor block, and sedation were assessed in the PACU at arrival and then every hour using the modified Hannallah pain scale, modified Bromage scale, and Ramsay sedation scale, respectively. The duration of analgesia (i.e., time between completion of caudal block and demand for first postoperative rescue analgesics) and the total requirement of rescue analgesics in 24 hours were observed. If the pain score was 4 or more, injection paracetamol 10 mg/kg was given as a rescue analgesic. Any side effects such as nausea and vomiting, urinary retention, respiratory depression, or bradycardia were noted.

Statistical analysis

Using G Power 3.1.9.6 version software for sample size calculation, and one-way analysis of variance (ANOVA) study, the total sample of 66 individuals achieved 80% power to detect differences among the mean duration of analgesia using an F-test with 0.05 significance level and an effect size of 0.4. For simplification of data collection, the sample size was reduced to 60.

SPSS version 23 (IBM Corp., Armonk, NY, USA) was used for data analysis. Group comparisons for continuously distributed data were made using an independent-sample t-test when comparing two groups. If data were non-normally distributed, appropriate non-parametric tests in the form of the Wilcoxon test were used. The chi-square test was used for group comparisons of categorical data. In case the expected frequency in the contingency tables was found to be <5 for >25% of the cells, Fisher’s exact test was used. Linear correlation between two continuous variables was explored using Pearson’s correlation (if the data were normally distributed) and Spearman’s correlation (for non-normally distributed data). Statistical significance was kept at p-values of <0.05.

## Results

There was no statistically significant difference in the demographic profile of the patients, distribution of the type of procedure, and duration of surgery (Table 1).

**Table 1:** Association of different groups with demographic parameters ^1^One-way analysis of variance; ^2^Fisher’s exact test; ^3^Kruskal-Wallis test; ^4^chi-square test. ASA: American Society of Anesthesiologists

Parameters	Group			P-value
	L (n = 20)	LD (n = 20)	LC (n = 20)	
Age (years)	3.40 ± 1.31	3.70 ± 1.42	3.85 ± 1.39	0.576^1^
Gender				0.322^2^
Male	20 (100.0%)	18 (90.0%)	20 (100.0%)	
Female	0 (0.0%)	2 (10.0%)	0 (0.0%)	
Weight (kg)	13.70 ± 4.03	13.00 ± 2.38	14.10 ± 2.77	0.505^3^
ASA grade (I)	20 (100.0%)	20 (100.0%)	20 (100.0%)	1.000^4^
Procedure				1.000^2^
Herniotomy	16 (80.0%)	16 (80.0%)	15 (75.0%)	
Orchidopexy	4 (20.0%)	4 (20.0%)	5 (25.0%)	
Onset of the block (minutes)	14.05 ± 1.70	12.30 ± 1.26	11.95 ± 1.23	<0.001^3^
Duration of surgery (minutes)	36.50 ± 3.66	35.65 ± 4.23	35.95 ± 4.84	0.748^3^

**Figure 2:**
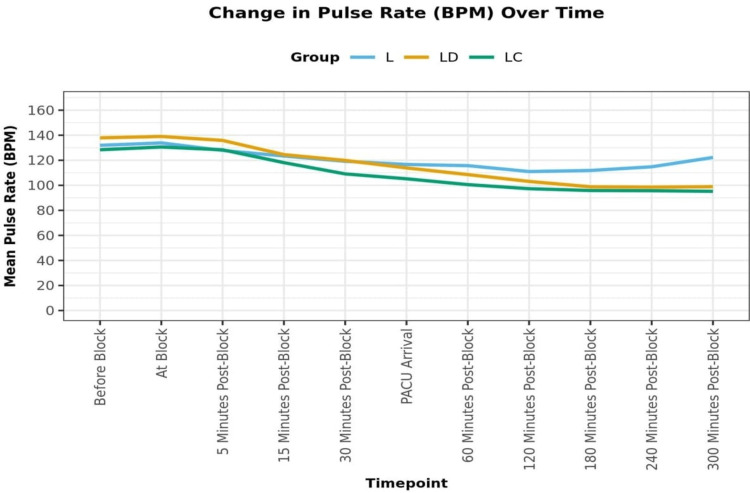
Change in pulse rate (beats per minute) over time.

There was a significant difference in pulse rate (beats per minute, BPM) and MAP (mmHg) over time between the three groups (p = <0.001). After the caudal block, there was a decrease in the pulse rate of all three groups, which was more in group LC than in group LD and the least in group L (Figure 2).

**Figure 3:**
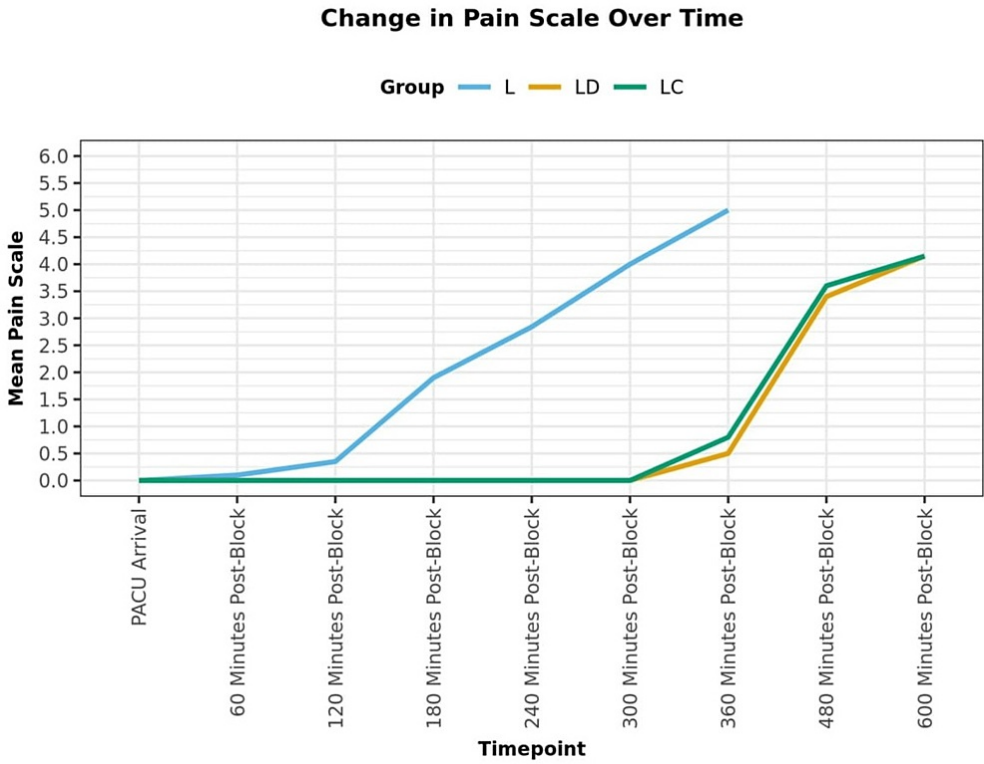
Change in pain scale over time.

The three groups differed significantly in terms of MAC. There was a declining trend in MAC values from 15 minutes to 30 minutes of intraoperative time. Mean MAC at 30 minutes was the lowest in group LD (0.69), followed by group LC (0.74) and group L (0.89). There was a significant difference in the trend of MAC over time between the three groups (p = 0.013). In groups LD and LC, the mean pain scale score increased from a minimum of 0.00 at the PACU arrival to a maximum of 4.15 at the 600-minute post-block timepoint. This change was statistically significant compared to group L (p = <0.001) (Figure 3).

The modified Bromage score was higher in both groups LD and LC compared to group L up to 120 minutes (p < 0.001). However, no significant residual motor block was present after 180 minutes of caudal block in any study group. Sedation score was 3 at 60 minutes post-block. All children were awake and alert 120 minutes post-block. The mean duration of analgesia in group LC was 492.00 ± 50.01 minutes, in group LD was 486.00 ± 54.71 minutes, and in group L was 291.00 ± 40.25 minutes. No significant difference was found between groups LC and LD (p = 0.989); however, a statistically significant difference was noted when compared to group L (p < 0.001) (Table 2).

**Table 2:** Duration of analgesia (minutes). SD: standard deviation; IQR: interquartile range

Duration of analgesia (minutes)	Group			Kruskal-Wallis test	
	L	LD	LC	χ^2^	P-value
Mean (SD)	291.00 (40.25)	486.00 (54.71)	492.00 (50.01)	42.184	<0.001
Median (IQR)	300 (300–300)	480 (465–495)	480 (480–495)		
Range	180–360	420–600	420–600		

There was a significant difference between the three groups in terms of the total dose of analgesics/24 hours (p <0.001), with the median total dose of analgesics being the highest in group L (2) (Table 3).

**Table 3:** Total number of doses of analgesics in 24 hours. SD: standard deviation; IQR: interquartile range

Total dose of analgesics/24 hours	Group			Kruskal-Wallis test	
	L	LD	LC	χ^2^	P-value
Mean (SD)	2.35 (0.49)	1.30 (0.47)	1.40 (0.60)	27.682	<0.001
Median (IQR)	2 (2–3)	1 (1–2)	1 (1–2)		
Range	2–3	1–2	1–3		

No episodes of clinically significant postoperative complications such as nausea, vomiting, respiratory depression, urinary retention, and pruritis were observed in any of the patients.

## Discussion

The main finding of our study was that the addition of dexmedetomidine 0.5 µg/kg to 0.25% levobupivacaine and the addition of clonidine 0.5 µg/kg to 0.25% levobupivacaine, when administered as caudal anesthesia in infraumbilical pediatric surgery, significantly prolongs the duration of postoperative analgesia (486 minutes and 492 minutes, respectively) when compared with 0.25% levobupivacaine alone (291 minutes). No significant motor blockade and side effects requiring intervention were observed.

Postoperative analgesia in pediatric patients needs special attention due to their inability to express the severity and type of pain. Adequate analgesia reduces the anxiety of the patient’s caretakers and helps in early recovery to perform daily activities and prevention of progression of acute postsurgical pain to chronic pain. Acute postoperative pain is reported to progress to chronic pain in 20% of children [6]. Therefore, it should be treated aggressively. Nowadays, multimodal analgesia is a preferable choice for perioperative pain relief. The caudal block is a relatively easy technique and is becoming an increasingly popular method for perioperative analgesia in pediatric infraumbilical surgeries.

A comparative study of intravenous paracetamol and caudal blocks to evaluate analgesic effects in pediatric patients undergoing surgery showed that postoperative analgesia was prolonged in the group receiving caudal blocks [7]. α2 adrenergic receptor agonists prolong the duration of action of levobupivacaine and improve the quality of analgesia by causing local vasoconstriction and increasing the potassium conductance in A-delta and C fibers. They also potentiate the action of local anesthetics by acting on alpha 2 receptors in the superficial laminae of the spinal cord and brainstem, or by indirectly activating spinal cholinergic neurons [8].

In our study, all three groups were comparable in terms of age, gender, weight, type, and duration of surgery. Previous studies have shown that the hemodynamic parameters remain stable intraoperatively and postoperatively and were comparable at all time groups after the caudal block [9,10]. These findings are consistent with the results of our study. A study reported that caudal anesthesia reduces the requirement for a maintenance dose of inhalational agents [11]. Kim et al. in their study found that caudal analgesia significantly reduces sevoflurane consumption in anesthetized children [12].

Previous studies have shown that the addition of adjuvants such as clonidine and dexmedetomidine to caudal levobupivacaine has resulted in overall lowering of pain scores. Potti et al. showed that the group receiving caudal levobupivacaine with clonidine had lower pain scores compared to caudal levobupivacaine alone or with intravenous clonidine [13]. Elfawal et al. reported that FLACC (face, legs, activity, cry, and consolability) pain scores were significantly lower in the early six hours in the caudal levobupivacaine with dexmedetomidine group compared to the group receiving fentanyl as adjuvant [10]. All these findings are comparable with the results of our study.

Evidence regarding a residual motor block is less clear. Previous studies have shown some degree of motor blockade after caudal block [14,15]. In our study, we found that even though residual motor blockade was present just after surgery, it went away after 180 minutes of caudal block. We observed that residual sedation was present 60 minutes after the block, which might be due to the effects of premedication and ketamine. As we used lower doses of both clonidine and dexmedetomidine, we found that there was no sedation and children were wide awake and alert by 120 minutes.

The mean duration of analgesia was the highest in group LC (492.00 ± 50.01 minutes), followed by group LD (486.00 ± 54.71 minutes), and was the lowest in group L (291.00 ± 40.25 minutes). The duration of analgesia in other studies varied between 210 minutes and 990 minutes [9,16]. In our study, we used a low dose (0.5m µg/kg) of adjuvants compared to other studies which used higher doses. This may be the reason for the difference in our results when compared to these studies. These wide differences might also be due to the use of premedication, local anesthetics, different doses of adjuvants, types of anesthesia techniques, different scales of pain assessment, and different statistical analyses. A study by Ram et al. with caudal levobupivacaine along with dexmedetomidine showed that the mean duration of analgesia was 467 ± 140 minutes compared to levobupivacaine alone (276 ± 99 minutes) [17]. Elfawal et al. in their study found that caudal with dexmedetomidine 1 µ/kg has a prolonged duration of analgesia (490.4 ± 13.6 minutes) compared to caudal levobupivacaine alone (321.8 ± 10.2 minutes).

Subramaniam et al. (2021) reported that the requirement for rescue analgesics was lesser in the group given caudal dexmedetomidine compared to caudal bupivacaine alone [18]. Their findings are similar to those of our study. However, we found no significant difference in the requirement of total dose of rescue analgesics between groups LD and LC as both provide the same duration of analgesia.

Because there is a paucity of literature on the efficacy of caudal blocks with such low doses of α2 agonists, we conducted this study to determine the minimum effective dose of α2 agonists barring side effects. We found that with the use of a low dose of adjuvants, the mean duration of analgesia achieved is acceptable for patients and parents along with the lesser duration of motor blockade, no sedation, and no urinary retention, as seen with higher doses of adjuvants.

Study limitations

The major limitations of this study were the relatively smaller sample size and a single hospital-based investigation, making it difficult to generalize the results to the study population. The distinction between sedation and analgesia is a challenge in the pediatric population as a pain-free child is comfortable and asleep. There may also be discordance between self-reporting and behavioral pain measures in children three to six years of age after recovery.

## Conclusions

The addition of α2 agonists such as clonidine or dexmedetomidine at a dose of 0.5 µg/kg as an adjuvant to caudal levobupivacaine (0.25%) at 1 mL/kg significantly prolongs the duration of opioid-free analgesia in children undergoing infraumbilical abdominal surgeries without prolonging the motor blockade and any side effects. Moreover, dexmedetomidine does not offer significant advantage over clonidine regarding the analgesia duration.
